# Author’s Reply to González-Mohíno et al. “A Pragmatic Approach to Resolving Technological Unfairness: The Case of Nike’s Vaporfly and Alphafly Running Footwear”

**DOI:** 10.1186/s40798-021-00379-7

**Published:** 2021-12-17

**Authors:** Bryce Dyer

**Affiliations:** Faculty of Science & Technology, Bournemouth University, Fern Barrow, Poole, UK

**To the Editor**,

I was pleased to see that my manuscript investigating the acceptability of the Nike Vaporfly and Alphafly running footwear generated a response [1]. This has been a controversial topic in competitive sport and such dialogue is helpful in enriching the debate. Their additional analysis detailed in their letter further provides support to the existing literature that the shoes design is performance enhancing, and I fully support that supposition. However, the authors of the letter have primarily stated two main concerns derived from the Dyer paper [2]. This response will address these and the conclusions made in the Rodrigo-Carranza et al. letter.

1. Performance progression in long-distance, such as the marathon and the half-marathon, has not changed

No claim was made in the Dyer paper that the progression in running distance records had remained unchanged. However, what was argued was that the use of the ‘Performance Improvement Index’ in the paper demonstrated that the index scores obtained since the shoes were introduced were not unusual when compared to the index scores seen in a range of other sports that had seen technological innovation [3]. It should also be noted that despite the performance increases that Rodrigo-Carranza et al. proposed, such changes were also deemed acceptable to the sport when its governing body implemented rules on footwear design in 2020 that then allowed any retrospective performances to stand (https://www.worldathletics.org/news/press-releases/modified-rules-shoes).

Nonetheless, an excellent point that Rodrigo-Carranza et al. highlighted for further investigation is whether distance running and its use of technology should be judged uniquely to that of other sports.

2. The shoes’ introduction has not obtained any greater change than the reasons attributed to any former record being broken.

This comment was made in the Dyer paper specifically in reference to the claim that the outlawing of the Nike shoes would somehow protect the legacy and history of the sport [4]. Indeed, by doing so would have arguably prevented a similar controversy to those experienced when full body suits were allowed in swimming [5]. It also assumes that footwear innovation has not occurred in distance running before but this has been indicated to not be the case [6]. Therefore it is not as simple to infer that the Nike shoes were a unique “technological revolution” when Rodrigo-Carranza et al. did not comment on the impact of other innovations that have occurred in the sports past and the impacts that these may have had on running performance trends. Ultimately though, this comment in the Dyer paper was merely intended to highlight that the Nike footwear case was not unique and that distance running was not somehow immune to technological interventions until their arrival.

3. The probable unfairness of Nike Vaporfly/Alphafly shoes

In their conclusion, Rodrigo-Carranza et al. stated that they felt the Nike shoes are “probably unfair”. They suggested this was due to “the greater improvements the shoes provide when compared to the years prior of the technological revolution”. Holowchak highlighted the relativity and subjectivity of the concept of *fairness* with sports technology [7]. It was demonstrated that such judgements may need to rely on some contribution of qualitative discourse rather than assuming an arbitrary increase in performance then equates directly to being deemed ‘unfair’. This was the reason why the Dyer paper utilised a broad 11-item analysis which had been based upon a framework derived from 31 reported cases of sports technology controversy [8] and then proposed an overall conclusion ‘taken on balance’. However, Rodrigo-Carranza et al. only seemingly focused on just one of the framework points in their letter (being that of ‘do the Nike Vaporfly/Alphafly shoes provide an unfair advantage’?) and did not comment on the others. I would argue to determine the fairness of a technology based upon one of these items alone when so many more have been indicated in the wider literature [8] is an oversimplification of the issues that surround the adoption of any technological innovation in a sport.

Nonetheless, I applaud the approach taken by Rodrigo-Carranza et al. to consider a larger sample of athletes and performances using ‘season bests’ and ‘best times’ to provide a more societal view of elite sport than the Dyer paper which opted to focus on absolute best changes in the world records. I certainly look forward to reading their investigation of the Nike footwear controversy in a peer-reviewed publication in the future.

## Data Availability

Not applicable.
